# *IFNL4* Genotypes Predict Clearance of RNA Viruses in Rwandan Children With Upper Respiratory Tract Infections

**DOI:** 10.3389/fcimb.2019.00340

**Published:** 2019-10-04

**Authors:** Belson Rugwizangoga, Maria E. Andersson, Jean-Claude Kabayiza, Malin S. Nilsson, Brynja Ármannsdóttir, Johan Aurelius, Staffan Nilsson, Kristoffer Hellstrand, Magnus Lindh, Anna Martner

**Affiliations:** ^1^TIMM Laboratory, Department of Infectious Diseases, Sahlgrenska Cancer Center, Institute of Biomedicine, Sahlgrenska Academy, University of Gothenburg, Gothenburg, Sweden; ^2^Pathology Unit, Department of Clinical Biology, School of Medicine and Pharmacy, University of Rwanda, Kigali, Rwanda; ^3^Department of Virology, Institute of Biomedicine, Sahlgrenska Academy, University of Gothenburg, Gothenburg, Sweden; ^4^Department of Pediatrics, School of Medicine and Pharmacy, University of Rwanda, Kigali, Rwanda; ^5^Department of Mathematical Sciences, Chalmers University of Technology, Gothenburg, Sweden; ^6^Department of Laboratory Medicine, Institute of Medicine, Sahlgrenska Academy, University of Gothenburg, Gothenburg, Sweden

**Keywords:** interferon lambda, infection, single nucleotide polymorphisms, *rs12979860*, dinucleotide polymorphisms, *rs368234815*, upper respiratory tract, RNA virus

## Abstract

Polymorphisms in the interferon lambda gene locus (*IFNL*) such as the *IFNL4* genetic variants *rs12979860* and *rs368234815* are predictive of resolution of hepatitis C virus infection, but information about the impact of these variants in other infections is scarce. This study aimed at determining the potential impact of *IFNL4* variation for the clearance of respiratory tract pathogens in Rwandan children (≤5 years old, *n* = 480) seeking medical care for acute respiratory infections. Nasopharyngeal swabs were retrieved from all children at the first hospital referral and from 161 children at follow-up visits 2 weeks later. The swabs were analyzed for pathogens by real-time PCR and for host cell *IFNL4* genotype at *rs12979860* and *rs368234815*. Approximately 1/3 of the children were homozygous for the *rs12979860* T allele and the *rs368234815* ΔG allele, which are overrepresented in subjects of African descent. These *IFNL4* variants were significantly associated with reduced clearance of RNA viruses. Our results suggest that *IFNL4* genotypes that are common among subjects of African descent may determine inefficacious clearance of RNA viruses from the respiratory tract.

## Introduction

In humans, the type III family of interferons (interferons lambda, IFN-λ) comprises four proteins (IFN-λ1-4) (Hemann et al., 2017). IFN-λs share several features of the type I and type II IFNs, including antiviral and immunoregulatory properties (Li et al., 2009; Griffiths et al., 2015; Syedbasha and Egli, 2017; Zanoni et al., 2017) and signal via a unique extracellular receptor complex composed of an IFN-λR1 chain and an IL-10R2 chain preferentially expressed by epithelial cells (Sommereyns et al., 2008; Mordstein et al., 2010). IFN-λs have been ascribed a role in anti-microbial defense in the respiratory and gastrointestinal tracts (Khaitov et al., 2009; Mordstein et al., 2010; Davidson et al., 2016; Hemann et al., 2017).

Human IFN-λs are encoded by the *IFNL* gene locus on chromosome 19 (Kotenko et al., 2002; Sheppard et al., 2002; Prokunina-Olsson et al., 2013). Polymorphisms in *IFNL4*, including *rs12979860* and *rs368234815*, predict viral clearance in subjects infected with hepatitis C virus (HCV) (Prokunina-Olsson, 2019). Carriers of C at *rs12979860* or TT at *rs368234815* are thus more likely to resolve primary or chronic HCV infection than those carrying *rs12979860* T or *rs368234815* ΔG alleles (Lindh et al., 2011; Griffiths et al., 2015; Prokunina-Olsson, 2019). The unfavorable ΔG allele at *rs368234815* forms an open reading frame in *IFNL4*, and carriers of TT thus do not express functional IFN-λ4 (Prokunina-Olsson et al., 2013). Details regarding the mechanisms that link variation in *IFNL* to clearance of HCV infection remain largely unknown (Rembeck and Lagging, 2015; Onabajo et al., 2019). The frequency of the unfavorable *IFNL4* genotypes is several-fold higher among Africans (35–40% TT at *rs12979860*) than among Esat-Asians and Caucasians (10–15% TT) (Ge et al., 2009; Indolfi et al., 2014; The 1000 Genomes Project Consortium, 2015). These racial differences largely mirror the likelihood of resolution of HCV infection, which is considerably lower in subjects of African descent than in Caucasians (Indolfi et al., 2014; Roberts et al., 2014; Griffiths et al., 2015).

This study aimed at determining the potential impact of *IFNL* genetic variation for the resolution of respiratory tract infections in a cohort of Rwandan children. Our results imply that carriers of *rs12979860* TT and *rs368234815* ΔG/ΔG genotypes show reduced clearance of RNA viruses from the upper respiratory tract.

## Methods

### Patients

Patients were recruited at five health centers, two district hospitals and one referral teaching hospital located in Kigali City and in the Southern Province of Rwanda. Children ≤5 years old (*n* = 480) with respiratory symptoms of not more than 5 days duration were eligible for inclusion in this study, which was conducted between 2009 and 2012. Sociodemographic and clinical data were collected using a predesigned questionnaire. Nasopharyngeal swabs were retrieved from all patients at the initial visit and from 161 cases at a 14-day follow-up. Specimens were shipped to the Department of Virology at University of Gothenburg for analysis of microbial content along with genotyping of germline *rs12979860* (CC, CT or TT) and *rs368234815* (TT/TT, TT/ΔG or ΔG/ΔG).

### Detection of Pathogens and Genotyping at *IFNL*

Nucleic acid from a 200 μL specimen of nasopharyngeal swabs was extracted in a MagNA Pure LC instrument (Roche Diagnostics, Mannheim, Germany) using the Total Nucleic Acid isolation kit. In brief, nucleic acids were eluted in 100 μl and 5 μL was used in 20 μL real-time PCR reactions containing Taqman Fast Virus 1-step Mastermix (ABI, for RNA targets) or Universal Mastermix (ABI, for DNA targets). The real-time PCR was performed using the ABI 7900 384-well system (Applied Biosystems, Foster City, CA). In these analyses, oligonucleotides targeting parainfluenzavirus 1–3, respiratory syncytial virus, metapneumovirus, influenza A virus, influenza B virus, coronaviruses (NL63, HKU1, OC43, 229E), enterovirus, rhinovirus, morbillivirus, bocavirus, adenovirus, *B. pertussis, S. pneumonia*, and *H. influenzae* were utilized. After a reverse transcription step at 46°C for 30 min followed by 10 min of denaturation at 95°C, 45 cycles of two-step PCR were performed (15 s at 95°C, 60 s at 58°C) (for details, see Andersson et al., 2014; Elfving et al., 2016).

Genotypes at *rs12979860* and *rs368234815* were determined on host DNA recovered from the nasopharyngeal swabs using a 5'nuclease assay with allele-specific TaqMan probes (Applied Biosystems, Carlsbad, CA, USA) in a reaction volume of 10 μl. Briefly, genomic DNA (3 μl) was added to a mixture (7 μl) of TaqMan genotyping master mix reagent and the TaqMan SNP genotyping assay mixture specific to the SNP being genotyped. *rs12979860* genotyping was performed using a predesigned assay (Applied Biosystems, Carlsbad, CA, USA), while custom MGB probes (Applied Biosystems, Carlsbad, CA, USA) and primers (Sigma-Aldrich, St. Louis, MO, USA) were used for genotyping of *rs368234815* at concentrations of 200 and 900 nM, respectively. Primer and probe sequences are provided in Table S1. Assays were run on a 7500 Fast Real-Time PCR system according to the manufacturer's instructions (Applied Biosystems, Carlsbad, CA, USA), in the following steps: allelic discrimination pre-read, amplification, and allelic discrimination post-read. The amplification step comprised a 10 min AmpliTaq enzyme activation at 95°C followed by 40 cycles of 15 s denaturation at 95°C and 1-min annealing at 60°C. After allelic discrimination post-read, data were analyzed using the 7500 SDS software (Applied Biosystems, Carlsbad, CA, USA). Automatic allele calling was employed to define SNP genotypes. *rs12979860* genotyping was performed in 477 patients (99.2% of the study population) among whom 161 had follow-up nasopharyngeal swabs (with 159 samples available for genotyping). *rs368234815* genotyping was performed in 156 out of 161 patients with follow-up nasopharyngeal swabs. Examples of allelic discrimination plots for IFNL4 *rs12979860* and *rs368234815* are shown in Figures S1 and S2, respectively.

### Data Management and Statistical Analysis

Sociodemographic and clinical data were analyzed using the statistical package for social sciences (SPSS) version 25.0 (IBM Corporation, New York, USA). Graphs were generated by the GraphPrism 8 software (GraphPad Software, Inc., CA 92037, USA). The chi-square test for trend or logistic regression were employed to relate genotypes to binary responses. Genotypes were coded 0, 1, or 2 according to favorable allele count. Fisher's exact test was employed when genotypes were collapsed into two groups.

### Ethical Considerations

The study was approved by the Ethics Committee of the National University of Rwanda and by the Regional Ethics Review Board in Gothenburg, Sweden (approval no. 052-08). Written informed consent was obtained from a caregiver for each child included in the study.

## Results

### Patients, Disease Characteristics, and Pathogens

This study enrolled 480 Rwandan children ≤5 years old seeking medical care for acute upper respiratory infection. Patient characteristics, symptoms and the respiratory pathogens detected at diagnosis are shown in Table 1. Twenty-seven percent of patients were infants (<1 year of age), 51% were 1–3 years old and 22% 4–5 years old. The most common symptoms at referral were cough (98%), fever (92%) and/or runny nose (78%). Twenty-nine percent of patients were admitted to hospital and 17% had received antibiotics 1 week prior to seeking medical care. Analyses of nasal swabs revealed that the vast majority of patients (85%) carried multiple pathogens. *Streptococcus pneumoniae* was the most common pathogen (detected in 82%) followed by *Haemophilus influenzae* (73%), rhinovirus (38%), enterovirus (21%), respiratory syncytial virus (18%), *Bordetella pertussis* (10%), and adenovirus (9%) (Table 1).

**Table 1:** Patients and disease characteristics.

**Characteristics**	***n***	**%**
**Gender (*****n*** **=** **471)**		
Male	256	54.4
Female	215	45.6
**Age (years**, ***n*** **=** **477)**		
<1	130	27.3
1–3	241	50.5
>3–5	106	22.2
History of atopy (*n* = 159)	25	15.7
Fever (*n* = 161)	148	91.9
Cough (*n* = 161)	158	98.1
Sore throat (*n* = 158)	10	6.3
Running nose (*n* = 161)	126	78.3
Dyspnea (*n* = 161)	5	3.1
Hospital admission (*n* = 438)	91	28.8
**Outcome (*****n*** **=** **212)**		
Recovered	210	99.1
Deceased	2	0.9
**Pathogens at baseline (*****n*** **=** **480)**		
**ss(+)RNA viruses**^**a**^		
Enterovirus	102	21.2
Rhinovirus	181	37.7
Coronavirus	18	3.8
**ss(–)RNA viruses**^**b**^		
Influenza A virus	33	6.9
Influenza B virus	27	5.6
HPIV^c^	21	4.4
RSV^c^	85	17.7
Morbillivirus	7	1.5
HMPV^c^	23	4.8
**DNA viruses**		
Bocaparvovirus	4	0.8
Adenovirus	42	8.8
**Bacteria**		
*B. pertussis*	50	10.4
*H. influenzae*	348	72.5
*S. pneumoniae*	394	82.0
**Number of pathogens (*****n*** **=** **480)**		
Single pathogen	73	15.2
Multiple pathogens	407	84.8
**Followed-up cohort**		
Yes	161	33.5
No	319	66.5

a *ss(+), single-stranded(+)*.

b *ss(–), single-stranded(–)*.

c *HPIV, human parainfluenza virus; RSV, respiratory syncytial virus; HMPV, human metapneumovirus*.

### Distribution of *IFNL4* Genotypes vs. Initial Infection

*IFNL4* genotyping showed that 18% of patients carried the CC genotype at *rs12979860* while 49 and 33% of patients, respectively, carried CT and TT genotypes (Table 2). Fourteen percent of patients carried the TT/TT genotype at *rs368234815* while 51 and 35% of patients, respectively, carried TT/ΔG and ΔG/ΔG genotypes. The frequency of the favorable alleles was 42.6% for *rs12979860*-C allele and 39.7% for *rs368234815-*TT allele (Table 2). Variation in *IFNL4* did not significantly impact on the type of initial infection (Table S2) or viral or bacterial loads (Table S3).

**Table 2:** Distribution of *IFNL4* genetic variants.

***IFNL4* genetic variants**	***n***	**%**	**HWE (*P*)^**a**^**
***rs12979860*** **(*****n*** **=** **477)**			
**Genotypes**			0.94
CC	86	18.0	
CT	234	49.1	
TT	157	32.9	
**Allele frequency**			
C		42.6	
T		57.4	
***rs368234815*** **(*****n*** **=** **156)**			
**Genotypes**			0.38
TT/TT	22	14.1	
TT/ΔG	80	51.3	
ΔG/ΔG	54	34.6	
**Allele frequency**			
TT		39.7	
ΔG		60.3	

a *If P ≥ 0.05, genotype frequencies are consistent with Hardy-Weinberg equilibrium (HWE)*.

### Inferior Clearance of Respiratory RNA Viruses in Patients Carrying Unfavorable *IFNL4* Alleles

Clearance of all viral or bacterial pathogens was observed in 2/161 cases (1.2%). When comparing clearance within individual pathogen groups it was observed that 16 out of 18 patients cleared all DNA viruses (89%), 56/132 (42%) cleared all RNA viruses while 6/157 (4%) cleared all bacteria. Furthermore, 69% of patients had acquired a new pathogen, most commonly an RNA virus (52%), at the second round of sampling (Table S4).

**Figure 1:**
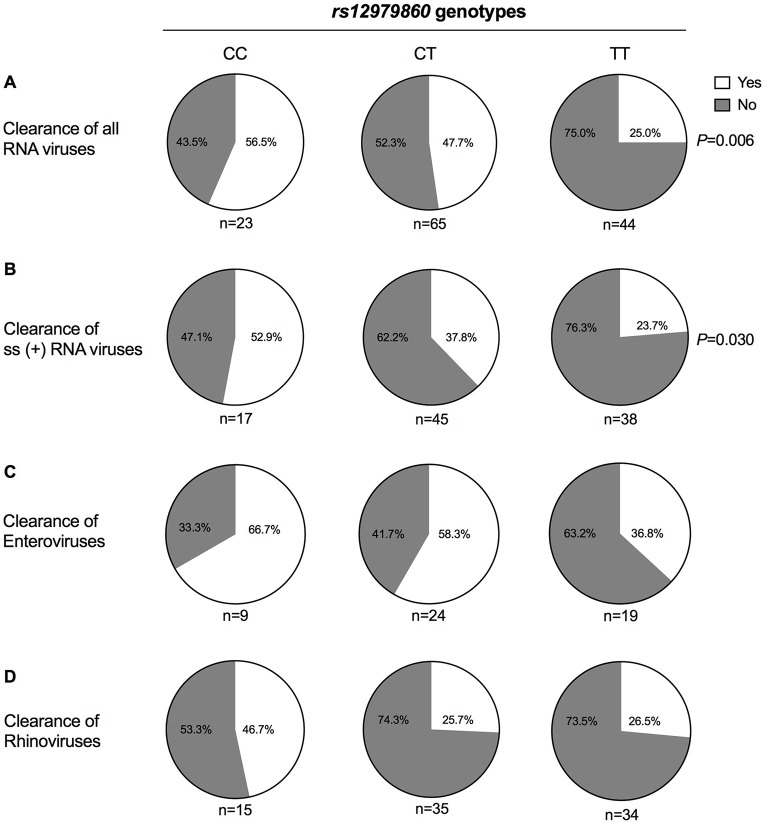
Respiratory RNA virus clearance vs. *rs12979860* genotypes. Nasopharyngeal swabs were collected from 161 Rwandan children aged 0–5 years seen at health facilities for respiratory infections at an initial visit and at a follow-up visit 2 weeks later. Swabs were analyzed for microbial content using real-time PCR. Two samples could not be analyzed, and 159 patients were thus stratified based on IFN-λ *rs12979860* genotype. Results show the proportion of patients within each genotype who cleared **(A)** all analyzed RNA viruses (i.e., enterovirus, rhinovirus, coronavirus, influenza A virus, influenza B virus, parainfluenzavirus, RSV, morbillivirus, and metapneumovirus), **(B)** all ss(+)RNA viruses (i.e., enterovirus, rhinovirus, coronavirus), **(C)** enterovirus, and **(D)** rhinovirus. Statistical analysis was performed using the Chi-square test for trend.

Analyses of the genotype at *rs12979860* vs. pathogen clearance revealed that patients carrying the TT genotype were over-represented among those who failed to clear RNA virus infections. Thus, while 57% of patients carrying the CC genotype and 48% of patients with CT genotype cleared all RNA viruses, this was only observed in 25% of patients carrying TT at *rs12979860* (*P* = 0.007, *OR* = 2.1 per C-allele, Figure 1A and Table 3). Moreover, 53% of patients with CC genotype cleared all single-stranded (+)RNA viruses, compared with 38% of patients with CT genotype and 24% of patients with TT genotype (*P* = 0.030, *OR* = 1.9 per C-allele, Figure 1B and Table 3). Adding age (*P* = 0.13) and gender (*P* = 0.7) did not improve the model for clearance of RNA viruses, but the association to *rs12979860* remained significant (*P* = 0.01, *OR* = 2.0). We did not observe a significant impact of *rs12979860* genotypes on the clearance of single-stranded (–)RNA viruses (*P* > 0.5).

**Table 3:** Association between *IFNL4 rs12979860* and *rs368234815* genotypes with clearance of upper respiratory tract pathogens.

***IFNL4* genotypes**	**Clearance of pathogens (number of cases)**											
	**All RNA viruses**		**ss (+) RNA viruses**		**Enteroviruses**		**Rhinoviruses**		**All DNA viruses**		**Bacteria**	
	**Yes**	**No**	**Yes**	**No**	**Yes**	**No**	**Yes**	**No**	**Yes**	**No**	**Yes**	**No**
***rs12979860***												
CC	13	10	9	8	6	3	7	8	1	0	3	25
CT	31	34	17	28	14	10	9	26	9	1	1	76
TT	11	33	9	29	7	12	9	25	7	1	2	51
*P*^a^	**0.007**		**0.03**		0.1		0.2		0.7		0.3	
OR (95% CI)^b^	2.1 (1.2, 3.5)		1.9 (1.1,3.5)		2.0 (0.9, 4.4)		1.5 (0.8, 2.8)		1.6 (0.1, 22)		2.0 (0.6,6.4)	
OR (95% CI)^b^, ^c^	2.0 (1.2, 3.5)		1.8 (1.0, 3.3)		2.1 (0.9, 5.0)		1.5 (0.8, 2.8)		1.6 (0.1, 24)		2.0 (0.6,6.7)	
***rs368234815***												
TT/TT	8	10	6	8	3	3	4	8	1	0	2	20
TT/ΔG	34	33	20	27	17	9	12	25	9	1	1	79
ΔG/ΔG	13	31	8	28	6	11	7	25	6	1	3	51
*P*^a^	0.1		0.08		0.2		0.3		0.7		0.9	
OR (95% CI)^b^	1.6 (0.9, 2.7)		1.8 (0.9, 3.3)		1.8 (0.7, 4.4)		1.4 (0.7, 2.8)		1.8 (0.1, 26)		1.1 (0.3, 3.7)	
OR (95% CI)^b^, ^c^	1.5 (0.9, 2.6)		1.6 (0.9, 3.1)		1.9 (0.7, 4.9)		1.4 (0.7, 2.8)		1.7 (0.1, 27)		1.1 (0.3, 3.8)	

a *P-values from logistic regression*.

b *OR, odds ratio per C allele for rs12979860 and TT allele for rs368234815; CI, confidence interval*.

c *Including age and gender as predictors*.

We also performed genotyping at *rs368234815* in patients with available follow-up data. As expected from earlier reports (Prokunina-Olsson, 2019), *rs12979860* and *rs368234815* were in strong linkage disequilibrium (*D*' = 0.998; *r*^2^ = 0.89, Table S5). In accordance, there was a trend toward improved clearance of all RNA viruses among carriers of TT allele at *rs368234815* (Table 3), and when TT allele carriers were compared to ΔG/ΔG the benefit for carriers of TT allele was significant (Fisher's exact test *P* = 0.039, *OR* = 2.1).

Similar albeit non-significant trends toward reduced clearance were observed in patients carrying TT/*rs12979860* and harboring the most commonly occurring RNA viruses (rhinoviruses and enteroviruses; Figures 1C,D and Table 3). *IFNL4* genotypes did not significantly predict reinfection with new viruses (Table S4). Two children, both carrying TT at *rs12979860*, died from infections; one had a rhinovirus infection and both carried *S. pneumoniae* and *H. influenzae*.

## Discussion

The type III or λ interferons are the most recently discovered members of the family of interferons. While details of the biological and pathophysiological role of IFN-λs are only partly understood, IFN-λs exert broad antiviral activity, similar to type I IFNs, and may participate in defense against viruses at epithelial surfaces (Mordstein et al., 2010). This notion is supported by the distribution of IFN-λ receptors, which is largely confined to epithelial surfaces, and by experimental studies implying a role for IFN-λ in protection against epithelial pathogens (Sommereyns et al., 2008; Prokunina-Olsson, 2019).

The most recently discovered member of the IFN-λ family, IFN-λ4, displays <30% amino acid identity with IFN-λ 1–3 but shares the antiviral activity of other IFN-λs (Kotenko et al., 2002; Prokunina-Olsson et al., 2013). The formation of IFN-λ4 is regulated by a dinucleotide frameshift variant, *IFNL4-*ΔG at *rs368234815*, which is located within exon 1 of *IFNL4* (Griffiths et al., 2015; Prokunina-Olsson, 2019). Subjects carrying the ΔG allele at *rs368234815* are able to synthesize the IFN-λ4 protein whereas those carrying TT/TT at this locus do not. An additional site of genetic variation, *rs12979860*, is located within intron 1 of *IFNL4* (Ge et al., 2009; Thomas et al., 2009)*. rs368234815* and *rs12979860* are in strong linkage disequilibrium (LD), i.e. inherited together, in particular in subjects of East Asian and Caucasian ancestry (Ge et al., 2009; Prokunina-Olsson, 2019). The genotypes at *rs12979860* and *rs368234815* strongly predict the outcome of HCV infection. Patients carrying T at *rs12979860* or ΔG at *rs368234815* thus are less likely to resolve untreated HCV infection and show markedly reduced clearance of HCV-RNA during treatment (Lindh et al., 2011; De Nicola et al., 2012; Rembeck and Lagging, 2015; Prokunina-Olsson, 2019).

These findings imply a role for IFN-λ4 in the defense against RNA viruses, but the results are counter-intuitive as subjects who cannot generate IFN-λ4, an antiviral protein, show improved outcome of HCV infection. The mechanisms that explain the apparent disadvantage of the formation of IFN-λ4 in defense against HCV remain to be defined. A commonly forwarded hypothesis takes the finding that induction of interferon-stimulated genes (ISGs) may render cells refractory to type I interferons into account. The formation of IFN-λ4, a strong inducer of ISG, may thus compromise the anti-viral defense exerted by type I IFNs. The hypothesis is bolstered by studies showing that the intrahepatic expression of ISGs is elevated in HCV-infected patients carrying unfavorable *IFNL4* genotypes and by reports showing that the expression of several ISGs heralds unfavorable outcome of HCV infection (Onabajo et al., 2019).

The results of this study suggest that the TT genotype at *rs12979860* as well as the ΔG/ΔG genotype at *rs368234815* herald inefficient clearance of RNA viruses from the nasopharynx of Rwandan children seeking medical care for acute respiratory tract infection. Our results thus largely mirror those obtained in studies of the impact of *IFNL4* polymorphisms on the spontaneous or treatment-induced clearance of HCV-RNA. In accordance with earlier studies (Ge et al., 2009; Prokunina-Olsson, 2019), *rs12979860* and *rs368234815* were in LD, although the association was weaker than that reported in East Asian and Caucasian populations.

The unfavorable *IFNL4* variants are several-fold more common in Africans than in subjects of non-African descent (Indolfi et al., 2014; Griffiths et al., 2015; The 1000 Genomes Project Consortium, 2015; Prokunina-Olsson, 2019), and we speculate that the high frequency of unfavorable *IFNL4* variants may contribute to severe respiratory RNA virus infections among African children. The *IFNL4* genotype did not predict clearance of DNA viruses or bacteria, which may imply that *IFNL4* polymorphisms selectively or specifically regulates defense against RNA viruses. However, it should be considered that relatively few subjects in this cohort of patients were infected by DNA viruses and that the rate of clearance of bacteria was low. Confirmatory studies are required to establish the proposed association between *IFNL4* polymorphisms and clearance of respiratory RNA viruses, including studies in adult populations.

## Data Availability Statement

The datasets for this manuscript are not publicly available. Requests to access datasets should be directed to Dr. Maria Andersson, maria.andersson.3@gu.se.

## Ethics Statement

This study was carried out in accordance with the recommendations of National University of Rwanda Research Ethics Committee, Rwanda, and the Regional Ethics Review Board in Gothenburg, Sweden, with written informed consent from caregivers of all subjects. All caregivers gave written informed consent in accordance with the Declaration of Helsinki.

## Author Contributions

BR, MA, MN, JA, KH, ML, and AM initiated and designed the study. BR, MA, J-CK, MN, and BÁ acquired data. BR, MA, J-CK, MN, BÁ, JA, KH, ML, and AM organized the database. SN, BR, MN, and AM performed statistical analyses. BR, MN, SN, KH, and AM drafted the manuscript. All authors contributed to manuscript revision, read, and approved the submitted version.

### Conflict of Interest

The authors declare that the research was conducted in the absence of any commercial or financial relationships that could be construed as a potential conflict of interest.

## Supplementary Material

Figure S1 Allelic discrimination plot for IFNL4 rs12979860. Example showing genotype clusters CC (circle), CT (triangle), TT (diamond) and no template controls (square).

Figure S2 Allelic discrimination plot for IFNL4 rs368234815. Example showing genotype clusters TT/TT (circle), ΔG/TT (triangle), ΔG/ΔG (diamond), and no template controls (square). For three cases in this plate the genotype was not possible to determine (cross) due to low concentrations of genomic DNA. Genotyping was repeated for such cases using higher DNA concentrations.
