# Supplementary figures and images for: IFNL4 Genotypes Predict Clearance of RNA Viruses in Rwandan Children With Upper Respiratory Tract Infections
Source: Front Cell Infect Microbiol. 2019 Oct 4;9:340. doi: 10.3389/fcimb.2019.00340 (PMC6787560; doi:10.3389/fcimb.2019.00340)

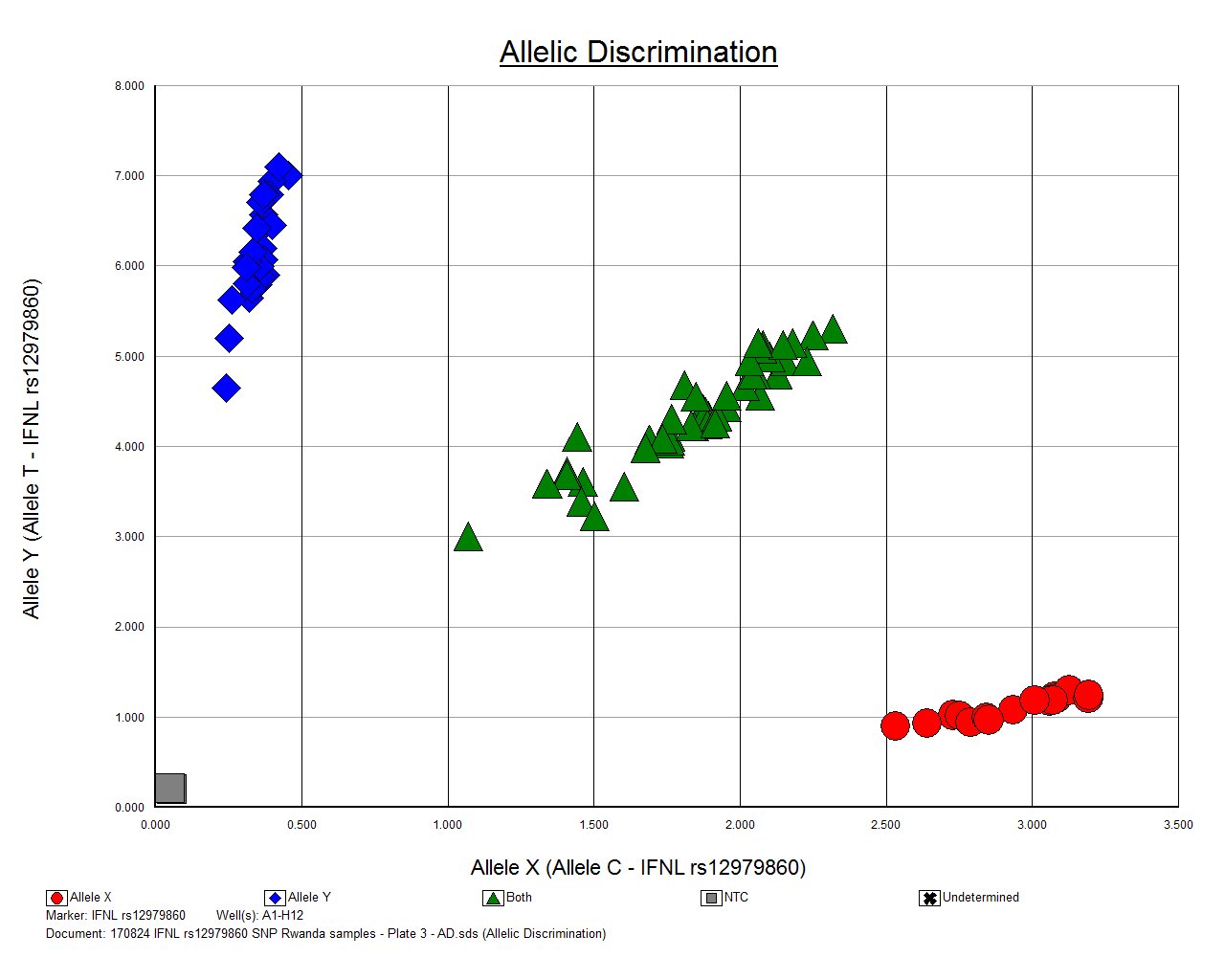

Supplement: Figure S1 — Allelic discrimination plot for IFNL4 rs12979860. Example showing genotype clusters CC (circle), CT (triangle), TT (diamond) and no template controls (square). [file Image_1.jpeg]

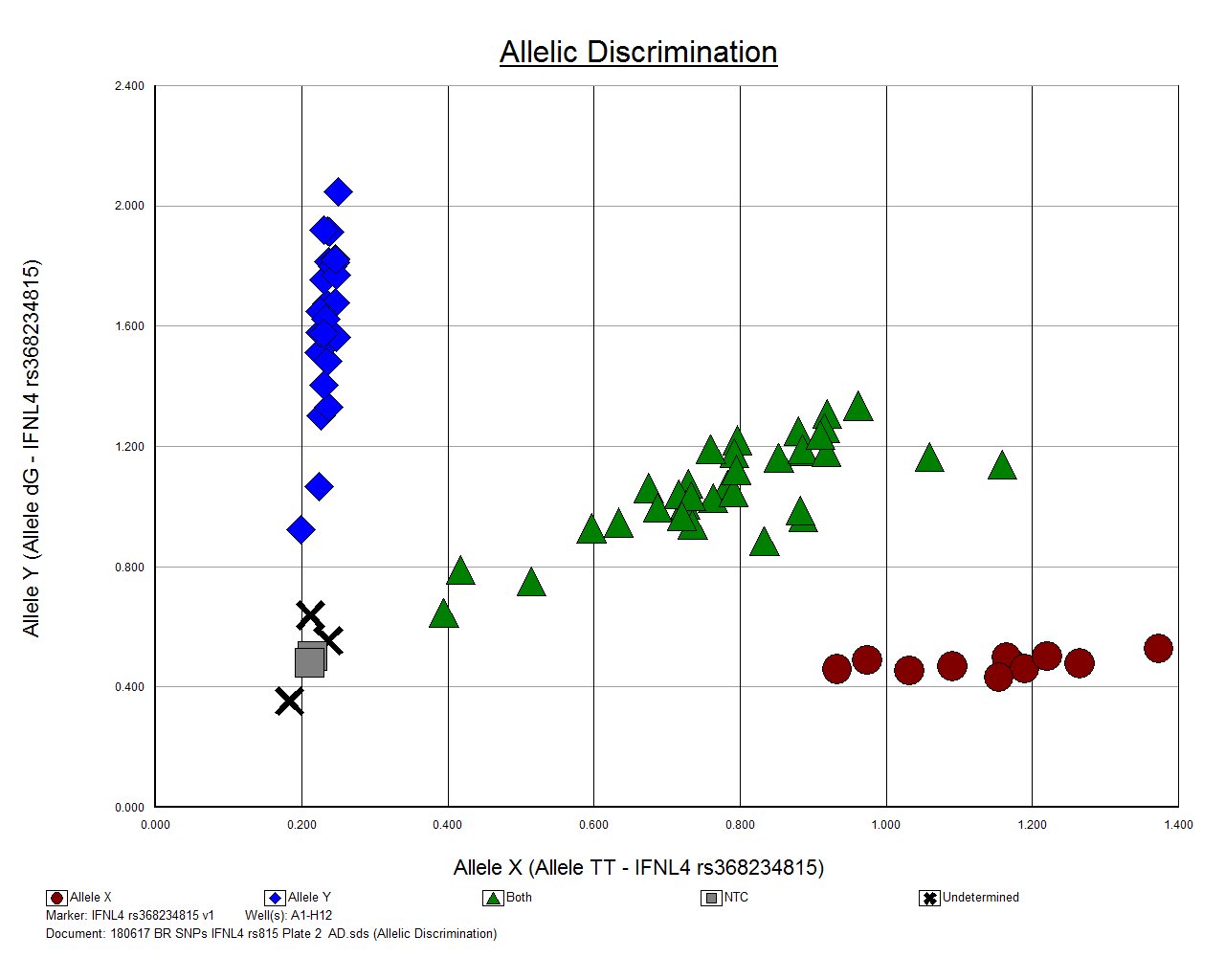

Supplement: Figure S2 — Allelic discrimination plot for IFNL4 rs368234815. Example showing genotype clusters TT/TT (circle), ΔG/TT (triangle), ΔG/ΔG (diamond), and no template controls (square). For three cases in this plate the genotype was not possible to determine (cross) due to low concentrations of genomic DNA. Genotyping was repeated for such cases using higher DNA concentrations. [file Image_2.jpeg]
