# Supplementary material for: IFNL4 Genotypes Predict Clearance of RNA Viruses in Rwandan Children With Upper Respiratory Tract Infections
Source: Front Cell Infect Microbiol. 2019 Oct 4;9:340. doi: 10.3389/fcimb.2019.00340 (PMC6787560; doi:10.3389/fcimb.2019.00340)
Supplement: Supplementary file 3 [file Table_1.docx]

**Table S1. Primers and probes**

| 1. **Pathogen isolation assays** | | | | |
| --- | --- | --- | --- | --- |
| **Target** | **Forward primer** | | **Reverse primer** | **Probe** |
| Parainfluenzavirus 1 | CAACAGGAAATCATGTTCTGTAATAGC | | TCACAGTGGGCAAGGAGCA | NED-CTGGTAACCCCTTGTTCCTG-MGB |
| Parainfluenzavirus 2 | GCATTTCCAATCTTCAGGACTATGA | | ACCTCCTGGTATAGCAGTGACTGAAC | JOE-CCATTTACCTAAGTGATGGAATCAATCGCAAA-BHQ1 |
| Parainfluenzavirus 3 | AAATGATCTGATTTATGCTTATACCTC | | TCAGGTACCAAGTCTGAGTTTACA | FAM-CGAGGTTGYCAGGATATAGGAAAATCA-BHQ1 |
| Respiratory syncytial virus | GCAAATATGGAAACATACGTGAACA | | GCACCCATATTGTWAGTGATGCA | FAM-CTTCACGAAGGCTCCACATACACAGCWG-BHQ1 |
| Metapneumovirus | ATGTCTCTTCAAGGGATTCACCT | | AMAGYGTTATTTCTTGTTGCAATGATGA | FAM-CATGCTATATTAAAAGAGTCTCARTAC-MGB |
| Influenza A virus | AAGACCAATCCTGTCACCTCTGA | | CAAAGCGTCTACGCTGCAGTCC | FAM-TTCACGCTCACCGTGCCMAGTG-BHQ1 |
| Influenza B virus | AAATACGGTGGATTAAATAAAAGCAA | | CCAGCAATAGCTCCGAAGAAA | VIC-CCATAGGAAATTGCC-MGB |
| Coronavirus NL63 | ACGTACTTCTATTATGAAGCATGATATTAA | | AGCAGATCTAATGTTATACTTAAAACTACG | NED-CCAAGGCTCCTAAACG-MGB |
| Coronavirus HKU1 | AAATGTGATCGTGCTATGCCAA | | CTTAACATAATAGCAACCGCCACA | VIC-CCTTGCGAATGAATG-MGB |
| Coronavirus OC 43 | CGATGAGGCTATTCCGACTAGGT | | CCTTCCTGAGCCTTCAATATAGTAACC | NED-CCTGGCACGGTACTC-MGB |
| Coronavirus 229E | CAGTCAAATGGGCTGATGCA | | AAAGGGCTATAAAGAGAATAAGGTATTCT | FAM-CCTGACGACCACGTTGT-MGB |
| Enterovirus | AGGTGYGAAGAGYCTATTGAGCTA | | GGACACCCAAAGTAGTCGGTTC | VIC-TCCGGCCCCTGAATG-MGB |
| Rhinovirus | GGTGTGAAGAGCCSCRTGTGCT | | GCAGGGTTTRGGTTAGCCRCATT | VIC-TCCGGCCCCTGAATG-MGB |
|  | GGTGTGAAGACTCGCATGTGCT | |  |  |
|  | GGGTGYGAAGAGYCTANTGTGCT | |  |  |
| Adenovirus | GCCACGGTGGGGTTTCTAAACTT | | GCCCCAGTGGTCTTACATGCACATC | HEX-TGCACCAGACCCGGGCTCAGGTACTCCGA-BHQ1 |
| Bocavirus | CGGGCTCATATCATCAGGAAC | | ATCACTTGGTCTGAGGTCTTCGA | FAM-CAATCAGCCACCTATC-MGB |
| Morbillivirus | CGATGACCCTGACGTTAGCA | | GCGAAGGTAAGGCCAGATTG | FAM-AGGCTGTTAGAGGTTGTCCAGAGTGACCAG-BHQ |
| 1. ***IFNL4* genotyping** | | | | |
| **Target** | | **Forward primer** | **Reverse primer** | **Probe** |
| *rs368234815* | | TGGGTCCTGTGCACGGTGAT | TCCCTCAGCGCCTTGGCA | VIC-ATCGCAGAAGGCC-MGB, FAM-ATCGCAGCGGCCC-MGB |
| *rs12979860* | | Sequences not provided by vendor (Applied Biosystems, Carlsbad, CA, USA) | | |
