# Supplementary material for: IFNL4 Genotypes Predict Clearance of RNA Viruses in Rwandan Children With Upper Respiratory Tract Infections
Source: Front Cell Infect Microbiol. 2019 Oct 4;9:340. doi: 10.3389/fcimb.2019.00340 (PMC6787560; doi:10.3389/fcimb.2019.00340)
Supplement: Supplementary file 4 [file Table_2.docx]

**Table S2. Respiratory pathogens detected at first visit *vs. rs12979860* genotypes**

| **Class of microbes** | **Microbe at baseline** | **Total** | **%** | ***rs12979860* genotypes (n=477)** | | | |
| --- | --- | --- | --- | --- | --- | --- | --- |
|  |  |  |  | **CC** | **CT** | **TT** | **P^a^** |
| **RNA viruses** | Yes | 325 | 68 | 59 | 159 | 107 | 0.96 |
|  | No | 152 | 32 | 27 | 75 | 50 |  |
| **DNA viruses** | Yes | 45 | 9 | 9 | 25 | 11 | 0.29 |
|  | No | 432 | 91 | 77 | 209 | 146 |  |
| **Bacteria** | Yes | 448 | 94 | 80 | 223 | 145 | 0.64 |
|  | No | 29 | 6 | 6 | 11 | 12 |  |

^a^ Chi-square test for trend.
