# Supplementary material for: IFNL4 Genotypes Predict Clearance of RNA Viruses in Rwandan Children With Upper Respiratory Tract Infections
Source: Front Cell Infect Microbiol. 2019 Oct 4;9:340. doi: 10.3389/fcimb.2019.00340 (PMC6787560; doi:10.3389/fcimb.2019.00340)
Supplement: Supplementary file 5 [file Table_3.docx]

**Table S3. Microbial load at first visit vs *rs12979860* genotypes**

| **Microbes** | **Number of cases** | **Microbial load (median)** | ***rs12979860* genotypes (n=477)** | | | |
| --- | --- | --- | --- | --- | --- | --- |
|  |  |  | **CC** | **CT** | **TT** | **P^a^** |
| **Enterovirus** | 102 | below | 4 | 30 | 18 | 0.20 |
|  |  | above | 11 | 23 | 16 |  |
| **Rhinovirus** | 179 | below | 14 | 48 | 29 | 0.42 |
|  |  | above | 15 | 36 | 37 |  |
| **Adenovirus** | 42 | below | 7 | 9 | 6 | 0.23 |
|  |  | above | 1 | 14 | 5 |  |
| ***H. influenzae*** | 347 | below | 35 | 80 | 59 | 0.69 |
|  |  | above | 26 | 92 | 55 |  |
| ***S. pneumoniae*** | 393 | below | 34 | 103 | 60 | 0.66 |
|  |  | above | 37 | 102 | 57 |  |

^a^ Chi-square test for trend.
