# Supplementary material for: IFNL4 Genotypes Predict Clearance of RNA Viruses in Rwandan Children With Upper Respiratory Tract Infections
Source: Front Cell Infect Microbiol. 2019 Oct 4;9:340. doi: 10.3389/fcimb.2019.00340 (PMC6787560; doi:10.3389/fcimb.2019.00340)
Supplement: Supplementary file 6 [file Table_4.docx]

**Table S4. New pathogens at the second visit vs *rs12979860* genotypes**

| **Class of microbes** | **New microbe** | **Total** | **%** | ***rs12979860* genotypes (n=159)** | | | |
| --- | --- | --- | --- | --- | --- | --- | --- |
|  |  |  |  | **CC** | **CT** | **TT** | **P^a^** |
| **RNA viruses** | Yes | 82 | 52 | 11 | 42 | 29 | 0.30 |
|  | No | 77 | 48 | 17 | 35 | 25 |  |
| **DNA viruses** | Yes | 13 | 8 | 2 | 4 | 7 | 0.23 |
|  | No | 146 | 92 | 26 | 73 | 47 |  |
| **Bacteria** | Yes | 44 | 28 | 7 | 20 | 17 | 0.48 |
|  | No | 115 | 72 | 21 | 57 | 37 |  |
| **Any microbe** | Yes | 110 | 69 | 15 | 54 | 41 | 0.05 |
|  | No | 49 | 31 | 13 | 23 | 13 |  |

^a^ Chi-square test for trend.
