# Supplementary material for: IFNL4 Genotypes Predict Clearance of RNA Viruses in Rwandan Children With Upper Respiratory Tract Infections
Source: Front Cell Infect Microbiol. 2019 Oct 4;9:340. doi: 10.3389/fcimb.2019.00340 (PMC6787560; doi:10.3389/fcimb.2019.00340)
Supplement: Supplementary file 7 [file Table_5.docx]

Table S5. Linkage disequilibrium* between *rs12979860* and *rs368234815*

| *rs12979860* genotypes | *rs368234815* genotypes | | | *Total* |
| --- | --- | --- | --- | --- |
|  | TT/TT | TT/ΔG | ΔG/ΔG |  |
| CC | 22 | 5 | 1 | 28 |
| CT | 0 | 75 | 2 | 77 |
| TT | 0 | 0 | 51 | 51 |
| Total | 22 | 80 | 54 | 156 |

* D’=0.998; r^2^=0.89
